# Microbial Life Deep Underfoot

**DOI:** 10.1128/mBio.03201-19

**Published:** 2020-01-28

**Authors:** Jay T. Lennon

**Affiliations:** a Department of Biology, Indiana University, Bloomington, Indiana, USA

**Keywords:** environmental microbiology, microbial ecology, soil microbiology

## Abstract

Soil is one of the most diverse microbial habitats on Earth. While the distribution and abundance of microbial taxa in surface soils have been well described, the phylogenetic and functional diversity of bacteria and archaea in deep-soil strata remains unexplored. Brewer et al. (mBio 10:e01318-19, 2019, https://doi.org/10.1128/mBio.01318-19) documented consistent shifts in the composition and genomic attributes of microbial communities as a function of depth in 20 soil pits that spanned a range of ecosystems across North America. The unique microorganisms found in deep soils appear to be adapted to conditions of low energy based on the recovery of genes that code for traits such as internal resource storage, mixotrophy, and dormancy.

## Abstract (precis)

Soil is one of the most diverse microbial habitats on Earth. While the distribution and abundance of microbial taxa in surface soils have been well described, the phylogenetic and functional diversity of bacteria and archaea in deep-soil strata remains unexplored.

## COMMENTARY

For more than a decade, microbiologists have been characterizing the taxonomic, phylogenetic, and functional diversity of soils. With modern sequencing technologies developed during this time, it is not uncommon for thousands of microbial taxa to be recovered from a gram of soil. The maintenance of this diversity is likely influenced not only by fluctuating environmental conditions that are typical of soils but also by the physical complexity created by pores and aggregates that govern interactions among microorganisms and other members of belowground food webs. Microbiologists have also described how soil microbial communities vary across the landscape. Patterns of diversity among geographically distant sites can be affected by dispersal limitation, but they may also reflect differences in how species respond to underlying variation in pH, temperature, moisture, and nutrients, all of which are influenced to various degrees by geology, land use, and climate. Despite these methodological and conceptual advances, our understanding of soil biodiversity remains incomplete because scientists tend to focus on the microorganisms that reside in the top few centimeters while neglecting those that live deeper below Earth’s surface.

**FIG 1:**
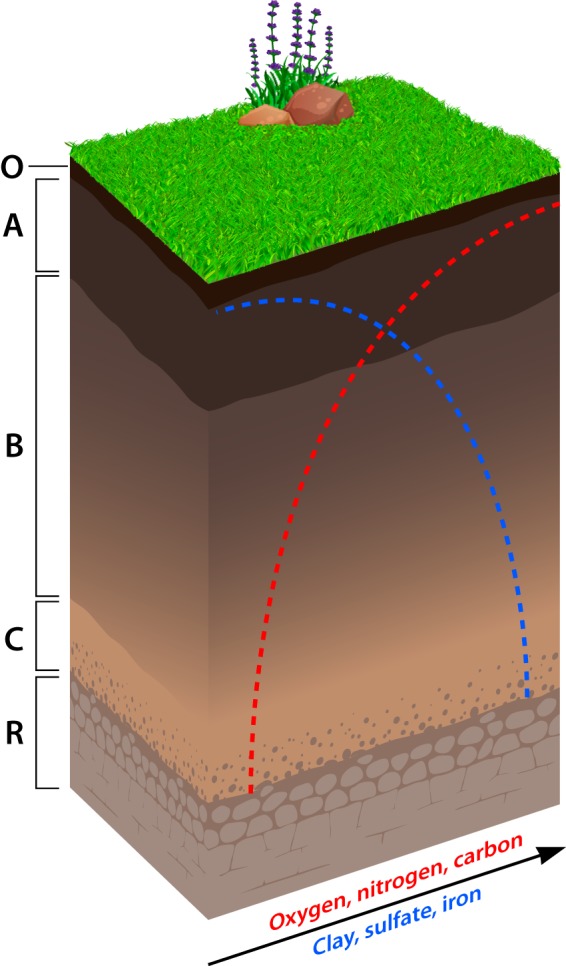
Typical profile depicting the distinct horizons that develop during soil formation. The O horizon is the surface layer containing nondecomposed plant material. The A horizon is the surface layer high in organic content and biological activity. The B horizon consists of subsoil with less organic material and higher concentrations of clays and minerals, including iron and aluminum oxides. The C horizon is substratum enriched in poorly weathered parent material, and the R horizon is bedrock representing the bottom of the soil profile. Despite these delineations, many soil properties change monotonically with depth depending on soil type, climate, and history.

To better understand the ecology of soil microorganisms, it is important to consider how soils are formed. It can take millennia or longer for soil horizons to fully develop. Newly formed soils are the product of weathering, along with the accumulation of organic matter derived from plant litter and root exudates that is subsequently modified by microorganisms. In these upper profiles, elements undergo rapid biogeochemical cycling owing to elevated temperatures and the tight metabolic coupling between plants and microorganisms. Moving into deeper horizons, soils exhibit dramatically different physical, chemical, and biological characteristics (Fig. 1). Changes in texture and structure are accompanied by lower redox potential, reduced availability of labile carbon, and the leaching of minerals due to the percolation of water through the soil profile. As a consequence, the supply of energy and nutrients required for the maintenance and growth of microorganisms becomes vanishingly small. While contending with these less-than-favorable environmental conditions, microbial communities in deep soils are also physically isolated from the more abundant and diverse regional pool of species at the surface. As a result, deep soils represent a unique habitat that likely constrains the composition and function of microbial communities.

To explore the diversity of deep-soil microbiomes, Brewer et al. (1) dug one-meter soil pits in 10 sites across the United States that make up part of the Critical Zone Observatory (CZO) network. In each pit, researchers took samples at the surface and then every 10 cm to document changes in soil properties and microbial communities across the depth profile. Approximately 20% of the variation in microbial composition could be explained by site location, which is not surprising given that samples were taken from contrasting ecosystems, including intensively managed farms, wet tropical forests, hot deserts, and subalpine forests. Although it explained far less variation (<1%), sampling depth in the soil horizon had a strong effect (*P*  < 0.001) on microbial composition. Compared to surface samples, microbial communities diverged in some cases by >75% through the soil horizon with the most dramatic changes occurring in the top 10 cm. These trends were driven by enrichment in deep soils of taxa belonging to the *Chloroflexi*, GAL15, *Nitrospirae*, *Euryarchaeota*, and *Dormibacteraeota* (formerly known as AD3). Collectively, the sequences from these five phyla accounted for 20 to 60% of all the 16S rRNA reads from the deep soils, reflecting the dominance of taxa that, in contrast, are numerically rare in surface soils.

The bacterial and archaeal taxa in deep-soil horizons lacked close matches to genomes in public databases while also belonging to lineages with very few cultured representatives. Thus, to gain insight into the traits and life history strategies that allow microorganisms to persist in deep soils, Brewer et al. (1) examined two draft genomes of *Dormibacteraeota* that were assembled from shotgun metagenomic sequences obtained from the soil pits. In contrast to a previous study that examined the surface soil from an Antarctic desert (2), *Dormibacteraeota* from deep soils lacked RuBisCO and hydrogenase genes. Combined with the recovery of high-affinity terminal oxidases and glycosyl hydrolases, the assembled genomes suggest that *Dormibacteraeota* are aerobic chemoorganoheterotrophs, even though deep-soil habitats likely experience periods of time where oxygen concentrations are reduced owing to lower atmospheric exchange and the lack of photosynthesis. However, the genomes also contained carbon monoxide (CO) dehydrogenase genes (*coxL*), which allow some bacteria to use CO as the sole carbon and energy source. This suggests that *Dormibacteraeota* in deep soils may meet their metabolic needs through a combination of trophic modes (i.e., mixotrophy).

As their name suggests, the *Dormibacteraeota* contain genes that may allow them to persist through unfavorable conditions by entering a reversible state of reduced metabolic activity. Dormancy, however, is not a cost-free strategy. Although not growing, cells must still energize their membranes, maintain osmotic equilibrium with their environment, and repair damaged cellular cargo (3). These maintenance demands are commonly met by drawing down on endogenous resource reserves. For example, *Dormibacteraeota* from deep soils possess genes that allow for the synthesis and degradation of glycogen, a storage compound that should prolong the life span of microorganisms while dormant. The *Dormibacteraeota* genomes also contained genes that are involved in the formation of endospores. This form of dormancy is best understood from the study of model organisms (e.g., *Bacillus* and *Clostridium*) in the *Firmicutes* where >150 genes are thought to be involved in sporulation (4). While some of these genes can be lost through relaxed selection, it appears that ∼30 sporulation genes are required to produce a viable spore (4). Brewer et al. (1) recovered only 15 and 19 sporulation genes in their two draft genomes (∼70% assembly), and some of the critical transcription factors that coordinate the complex developmental process were not found. Nevertheless, in a cultivation-independent assay, the authors demonstrated that *Dormibacteraeota* tolerated ethanol exposure, which was hypothesized to kill off vegetative cells but not spores.

The study by Brewer et al. (1) showcases a novel dimension of soil microbial biodiversity. While bacteria in deep soils are less abundant and active than assemblages found in surface soils, they nevertheless conduct unique biogeochemical processes that contribute to further development of the soil environment. Perhaps more intriguingly, their findings raise interesting questions about the constraints and adaptations to living in oligotrophic habitats, which may require a reevaluation of how important traits, like sporulation, are distributed among newly discovered bacterial phyla. The study sheds light on the mechanisms by which microorganisms persist in energy-limited “deep” environments, including soils and other subsurface ecosystems that are prevalent in our microbially dominated biosphere (5).
